# Levodopa-induced dyskinesia: interplay between the N-methyl-D-aspartic acid receptor and neuroinflammation

**DOI:** 10.3389/fimmu.2023.1253273

**Published:** 2023-10-04

**Authors:** Fanshi Zhang, Mei Liu, Jinmei Tuo, Li Zhang, Jun Zhang, Changyin Yu, Zucai Xu

**Affiliations:** ^1^ Department of Neurology, Affiliated Hospital of Zunyi Medical University, Zunyi, China; ^2^ The Collaborative Innovation Center of Tissue Damage Repair and Regeneration Medicine of Zunyi Medical University, Zunyi, China

**Keywords:** N-methyl-D-aspartate receptor, neuroinflammation, Parkinson’s disease, levodopa, levodopa-induced dyskinesia

## Abstract

Parkinson’s disease (PD) is a common neurodegenerative disorder of middle-aged and elderly people, clinically characterized by resting tremor, myotonia, reduced movement, and impaired postural balance. Clinically, patients with PD are often administered levodopa (L-DOPA) to improve their symptoms. However, after years of L-DOPA treatment, most patients experience complications of varying severity, including the “on-off phenomenon”, decreased efficacy, and levodopa-induced dyskinesia (LID). The development of LID can seriously affect the quality of life of patients, but its pathogenesis is unclear and effective treatments are lacking. Glutamic acid (Glu)-mediated changes in synaptic plasticity play a major role in LID. The N-methyl-D-aspartic acid receptor (NMDAR), an ionotropic glutamate receptor, is closely associated with synaptic plasticity, and neuroinflammation can modulate NMDAR activation or expression; in addition, neuroinflammation may be involved in the development of LID. However, it is not clear whether NMDA receptors are co-regulated with neuroinflammation during LID formation. Here we review how neuroinflammation mediates the development of LID through the regulation of NMDA receptors, and assess whether common anti-inflammatory drugs and NMDA receptor antagonists may be able to mitigate the development of LID through the regulation of central neuroinflammation, thereby providing a new theoretical basis for finding new therapeutic targets for LID.

## 1 Introduction

Parkinson’s disease (PD) is a common neurodegenerative disorder that affects middle-aged and elderly populations and is characterized by an insidious onset and slow progression. Its main clinical manifestations are resting tremor, bradykinesia, rigidity, and postural gait disorders (1). The main pathological change underlying PD is the degenerative death of dopaminergic neurons in the midbrain substantia nigra, which results in a significant decrease in striatal dopamine and thereby causes disease (2, 3). The exact etiology of this pathological change remains unclear, but genetic factors, environmental factors, aging, oxidative stress, and mitochondrial dysfunction have all been suggested to be involved in the degenerative death of dopaminergic neurons in PD (2, 4, 5). Drug therapy is the primary treatment for patients with PD, and levodopa (L-DOPA) is still the most effective drug (5), despite its side effects(6). Most patients with PD undergoing long-term dopamine drug treatment develop motor complications after an average of 6.5 years, the most common of which is L-DOPA-induced dyskinesia (LID) (7, 8, 9). LID manifests in abnormal movements such as stereotypic, choreiform, and throwing movements as well as in dystonia that mainly involves the head, face, limbs, and trunk and greatly affects the quality of life of patients with PD (9). The exploration of non-dopaminergic causative factors and the question of how L-DOPA drugs can be combined with other drugs that reduce these side effects have therefore attracted the attention of many scientists. The role of the neuroimmune inflammatory response in the pathogenesis of LID has also become the focus of research in recent years. In experimental work in animal models, we have identified that the release of neuroinflammatory factors, the activation and subtype transformation of microglia, and the activation of astrocytes all lead to the degenerative death and loss of dopaminergic neurons (10–12). The loss of dopaminergic neurons causes PD, and the mechanisms by which neuroinflammation may be involved in PD and LID are unknown and may be different (13, 14). Current preclinical studies support a role for neuroinflammation in LID, and it is not possible to determine whether exacerbation of neuroinflammation may contribute to the development of LID in patients with PD (15, 16). As patients with PD are often treated with L-DOPA in the later stages of the disease, it cannot be ruled out that this treatment may be related to neuroinflammation (17, 18). The mechanism of how L-DOPA therapy may augment the neuroinflammatory response, and how this may affect LID outcomes, remains to be studied (14, 16). A link between neuroinflammation and the N-methyl-D-aspartic acid receptor (NMDAR) has been found (19, 20), namely the activation of glial cells and the release of inflammatory factors such as tumor necrosis factor-α (TNF-α), interleukin-1β (IL-1β), inducible nitric oxide (NO), nitric oxide synthase (iNOS), and chemokines, which regulate the release of glutamic acid (Glu) from presynaptic neurons and the expression of Glu receptors in postsynaptic neurons (21, 22). NMDARs are common ionotropic Glu receptor channels, and expression and phosphorylation of their subunits GluN1 and GluN2 can contribute to the development of LID by regulating synaptic plasticity (23, 24), suggesting that the interaction between neuroinflammation and NMDARs may have an important role in the progression of LID. Synaptic plasticity refers to the regulation of the strength of synaptic connections (25, 26), by means of formation, elimination, enhancement, and weakening (25, 27). L-DOPA can lead to the development of LID through alterations in synaptic plasticity at the neurobiological level in the context of the pathologic changes underlying PD (25, 28). The corticostriatal synapses physiologically can undergo long-term potentiation (LTP), long-term depression (LTD) and depotentiation (“de-enhancement”) (27, 28). Normally, signals from the cortex reach the striatum, where they are filtered and integrated via LTP and LTD, and appropriate signals are exported from the basal ganglia to regulate movement and learning (27). In rodent models of PD, the cortex still sends signals to the striatum, but the striatum loses LTP and LTD, hence the ability to screen and integrate these signals and thus failing to appropriately regulate the output signals from the basal ganglia (26). In long-term L-DOPA-treated rodent models of PD, striatal synapses lose their ability to “de-enhance,” leading to uncontrolled enhancement in the pathway, which further contributes to the development of LID (26, 27). In this article, we review the possible mechanisms through which the interplay of NMDARs and neuroinflammation is involved in LID (29).

## 2 Overview of LID

As populations are aging, the global numbers of people with PD are increasing, placing a heavy financial and emotional burden on individuals, families, and society at large (30, 31). L-DOPA is currently recognized as a drug that offers good symptom control in patients with PD, but most patients will develop LID after long-term application, with mechanisms that are complex and not yet understood. LID is characterized by choreiform movements, dystonia, tardive dyskinesia, or simple repetitive involuntary movements, the severity of which often correlates with the degree of degeneration of dopaminergic neurons. In recent years, new research on presynaptic mechanisms, changes in postsynaptic plasticity, γ-aminobutyric acid (GABA)-ergic and glutamatergic neurons, and non-dopaminergic regulatory factors, as well as microvascular permeability, have provided new ideas and directions for the study of LID (32, 33). The pathogenesis of LID is complex and unclear. In this article, we mainly chose to review the effects of the interaction between neuroinflammation and NMDARs on the synaptic plasticity of neurons and to explore the mechanism and treatment of its role in LID.

## 3 Role of NMDARs in LID

In patients with PD, dopamine depletion and long-term treatment with L-DOPA trigger adaptive changes in glutamatergic transmission from the cortex to the striatum, leading to abnormalities in striatal NMDAR function(34). NMDARs are voltage- and ligand-gated cation channels, and their intracellular subunit composition, distribution, and phosphorylation level are related to their function. In particular, phosphorylation is an important mechanism for regulating the transport and channel properties of NMDARs(35, 36). In the brain, GluN1 and GluN2 (GluN2A, GluN2B, GluN2C, GluN2D) are the predominant NMDAR subunits (37, 38). As a calcium channel, it is mainly located in the postsynaptic membrane and recognizes the neurotransmitter Glu, the binding of which causes the excitation of postsynaptic neurons (39–41). Over-stimulation of NMDARs leads to a large inflow of extracellular Ca^2+^, which in turn activates Ca^2+^-dependent enzymes involved in protein, nucleic acid, and phospholipid catabolism, and NO synthesis. This leads to cell membrane rupture and cytoskeletal changes in dopaminergic neurons in brain tissue, thereby causing dopaminergic neurons to die (42).

Abnormal NMDAR expression alters the synaptic plasticity of neurons in the striatal region and further imbalances the basal ganglia circuit, thereby inducing motor complications, most commonly LID (43–45). In experimental work on animal models, NMDAR signaling via the extracellular regulatory protein kinase (ERK1/2) and mitogen-activated protein kinase (MAPK) pathways regulates neuronal development and refinement of synaptic connections to regulate the progression of LID (46, 47). Phosphorylation levels of several NMDAR subunits are involved in changes in synaptic plasticity, such as the induction of LTP, and play a role in learning and memory formation (48). LTP responds to the storage of various types of information at the synaptic level and is inextricably linked to aspects of learning and memory formation (48). LTP can also be reversed to normal levels by low-frequency stimulation, a phenomenon known as synaptic de-enhancement. This bidirectional regulation of synaptic plasticity is important in regulating motor information storage in the basal ganglia (49). The bidirectional regulation and plasticity of striatal synapses in rats with LID are more severely impaired and the loss of this de-enhancement leads to the storage of abnormal redundant motor information, which triggers the production of redundant movements, a process that is largely dependent on NMDAR-mediated over-activation of LTP (27, 50, 51). Phosphorylation levels of the GluN1 subunit and its carboxy-terminal 890, 896, and 897 serine sites were found to be elevated in the striatum of rats with LID (52). The GluN2 subunit also has an important role in LID. Individuals with PD and LID induced by long-term treatment with L-DOPA exhibit increased expression of GluN2B compared to individuals without LID (53, 54). On the other hand, since the occurrence of LID is related to GluN2A expression and the phosphorylation of ERK/MAPK, the probability of LID in PD rats was reduced by a peptide penetrating the cell membrane and cutting off nine amino acids from the C-terminus of GluN2A, thus inhibiting the expression and phosphorylation of GluN2A (55). In addition, the massive release of Glu upon activation of NMDAR increases excitatory postsynaptic calcium currents, induces LTP, exacerbates abnormal behavior, and promotes the development of LID (56, 57).

Among the NMDAR subunit types expressed by striatal projection neurons, GluN1, GluN2A, and GluN2B are most commonly regulated by phosphorylation modifications; however, GluN2C and GluN2D expression have recently been found to be associated with the development of LID as well (58). The synaptic abundance of GluN2D subunits is selectively increased in the rat striatum, allowing for a dramatic increase in the binding of the postsynaptic protein scaffold PSD-95. PSD-95 expression controls LID via dopamine D1 receptor trafficking (59, 60). Aberrant levels of NMDAR expression and phosphorylation thus play an important role in LID progression and act primarily through the induction of LTP following NMDAR activation, which alters synaptic plasticity between neurons.

## 4 Role of microglia in LID

**Figure 1:**
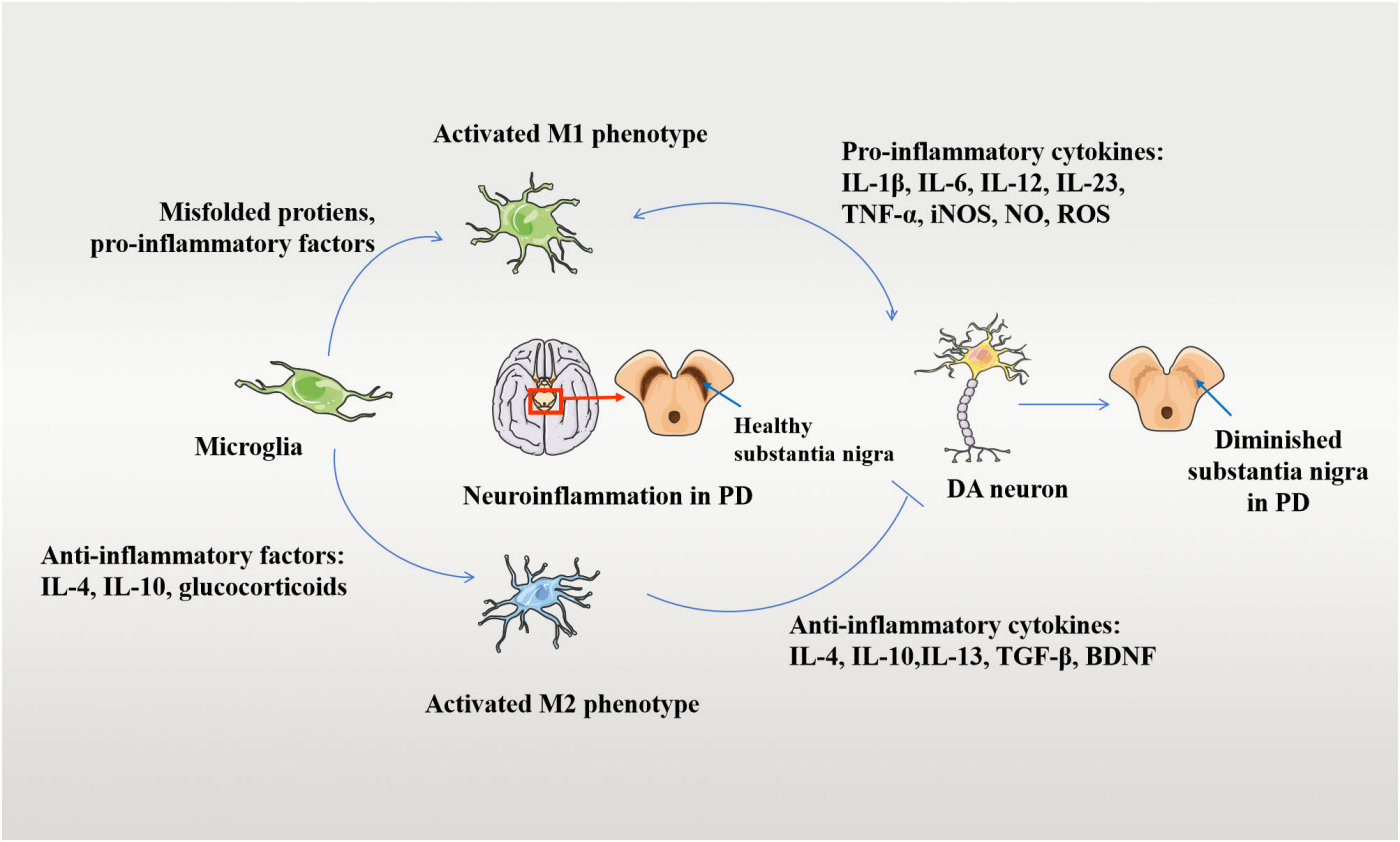
Mechanisms of damage to DA neurons by activated microglia. Microglia-mediated neuroinflammation and neuroprotective mechanisms in the pathogenesis of PD. Microglia turns into an activated M1 phenotype when exposed to stimuli such as infection, trauma, and intoxication. M1 phenotype microglia secrete pro-inflammatory factors, which further induce neuroinflammatory and neurotoxic mechanisms in the human brain through processes such as enhanced phagocytic activity and increased IL-1β, TNF-α, and ROS generation, damaging DAergic neurons. On the other hand, the presence and stimulation of anti-inflammatory factors can lead to an activated M2 phenotype. The neuroprotective mechanism of M2 microglia against PD involves the release of anti-inflammatory cytokines into the brain and the upregulation of neuroprotective trophic factors. The anti-inflammatory cytokines include IL-4, IL-10, and so on, which inhibit persistent neuroinflammation and consequently protect the DAergic neurons. Disease occurs when this balance in the organism is disrupted.

Microglia are the main immune cells in brain tissue and are an important component of neuroinflammation when activated. In the resting state, microglia are characterized by small cell bodies and elongated protrusions and are important in immune surveillance (61). Microglia are particularly susceptible to activation during brain injury or in response to inflammatory stimuli, which trigger the development of a neuroinflammatory response in brain tissue. Two main types of activated microglia have been identified: M1 and M2 (62, 63, 64). In basic experiments, M1 microglia release various neurotoxic mediators, including inflammatory cytokines IL-1β, TNF-α, iNOS, chemokines, NO, and oxygen radicals (62). These toxic mediators activate surrounding microglia and astrocytes in an autocrine or paracrine manner, creating a positive feedback loop that promotes the development of a neuroinflammatory response. These processes damage dopaminergic neurons and accelerate the progression of LID. M2 microglia are referred to as “anti-inflammatory” cells and mainly release anti-inflammatory mediators, including IL-4, IL-10, IL-13, and neuroprotective factors (brain-derived neurotrophic factor [BDNF], nerve growth factor [NGF], epidermal growth factor [EGF], glial-cell line-derived neurotrophic factor [GDNF]), which inhibit neuroinflammatory responses in brain tissue and promote tissue repair, thereby slowing disease progression (62). These phenomena are considered evidence for the activation of microglia in response to different environmental stimuli, through a series of organismic responses(64). **Figure 1** depicts the mechanism of microglia-mediated damage to dopaminergic neurons. Since microglia can switch between pro-inflammatory neurotoxic and anti-inflammatory neuroprotective phenotypes, this switch has been suggested to play a very important role in the development of LID (65, 66).

Microglia plays an important role in the regulation of synaptic plasticity. Recent and growing evidence suggests that changes in synaptic plasticity, particularly in LTP and LTD, may manifest through microglia (67). Although synaptic plasticity can manifest in many forms, the most carefully studied process with respect to learning and memory is LTP. LTP can be induced by the activation of NMDA-type Glu receptors, usually through the activity of presynaptic and postsynaptic neurons. Activated glial cells promote the development of LID by releasing inflammatory factors such as IL-1β as well as by upregulating NMDAR expression, which induces LTP and alters the plasticity of synapses between neurons. Current basic research has revealed that inflammatory factors released from microglia in brain tissue activate GluN2B, increase the levels of phosphorylated GluN2B subunit, and activate the downstream PKC/MEK/ERK signaling pathways, thus exacerbating the development of LID (16). The administration of continuous chronic lipopolysaccharide (LPS) stimulation to the rats with LID and analysis of the various NMDAR subtypes in their hippocampal and cortical regions revealed that chronic inflammatory stimulation further increases the release of inflammatory factors, resulting in increased expression of striatal GluN2B (16). It has therefore been speculated that the activation of microglia and astrocytes and the release of inflammatory factors in the striatum could influence the development of LID by affecting the activation or expression of this NMDAR. The release of inflammatory factors in an inflammatory rat model drove the activation of GluN2B, thereby allowing the Glu neurotransmitter to bind to GluN2B receptors, which leads to the opening of GluN2B receptor channels and the inward flow of Ca^2+^ ions (68, 69). These processes increase intracellular Ca^2+^ concentrations and subsequently trigger a series of biochemical reactions: G protein mediates the activation of phospholipase C (PLC), which catalyzes the hydrolysis of phosphatidylinositol (PI) to inositol triphosphate (IP3) and diacetylglycerol (DAG), which in turn activates protein kinase C (PKC) in the presence of Ca^2+^ (70–72). PKC not only enhances Ca^2+^-dependent Glu release and increases the sensitivity of the postsynaptic membrane to the transmitter, but also further enhances the Ca^2+^ influx into the cell via voltage-dependent channels. PKC phosphorylates MEK, which in turn affects the phosphorylation of ERK (73–75). In addition, there is evidence of enhanced phosphorylation of GluN1 in NMDARs in rats with inflammation, but there is a lack of definitive experimental results and literature on the specific signaling pathways involved in GluN1 phosphorylation (76). Thus, the neuroinflammatory response may have a further effect on LID by increasing the expression of subunits of striatal NMDARs (GluN1, GluN2) or by activating their function. In other words, whether neuroinflammation promotes and exacerbates LID by affecting the expression of GluN1 and GluN2 remains an open question. Only one basic experiment exploring these processes has ever been reported (6), and further studies are needed to elucidate the relationships between neuroinflammation, NMDARs, and LID and to provide a basis for finding therapeutic targets for LID.

## 5 Role of astrocytes in LID

**Figure 2:**
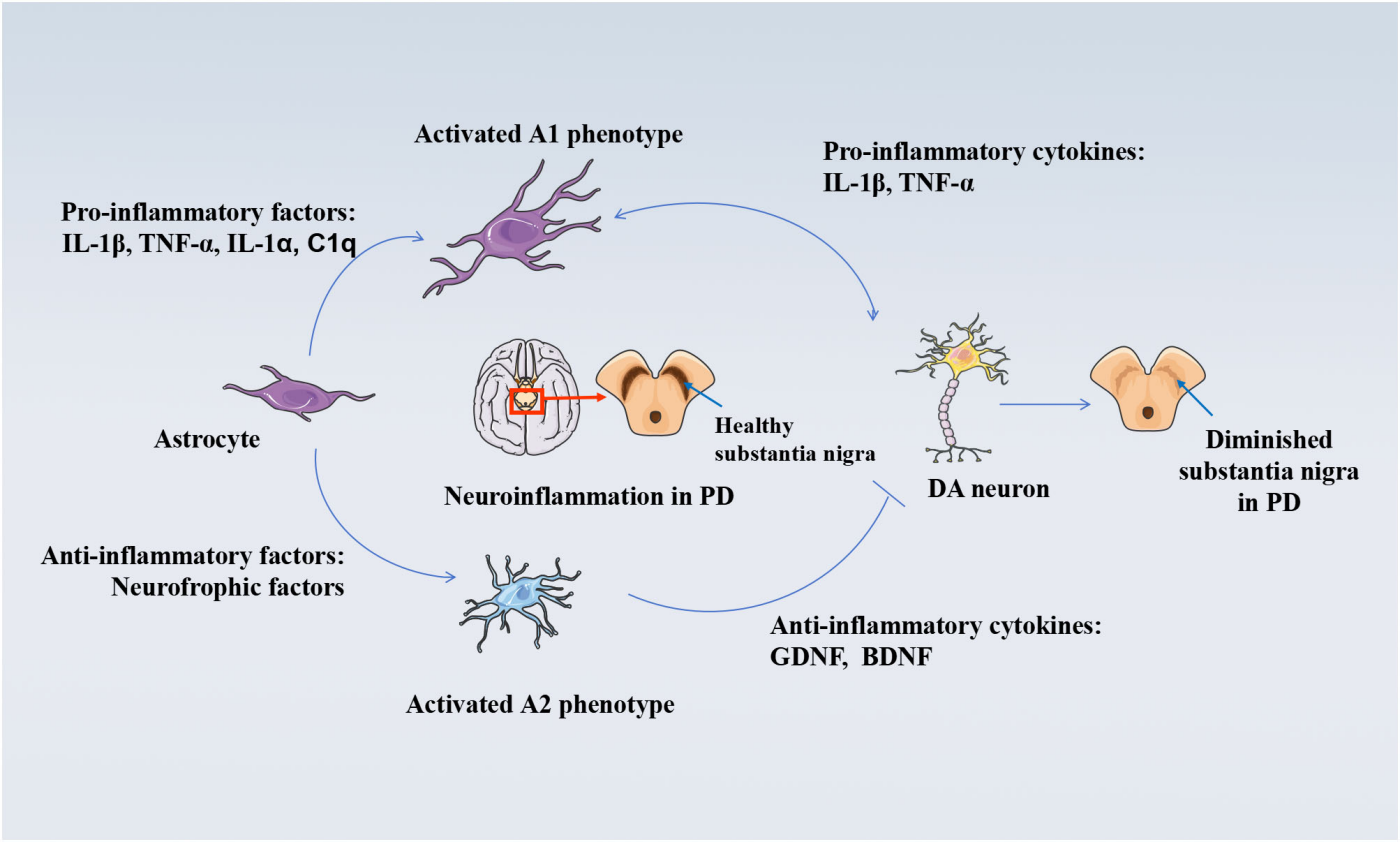
Mechanisms of damage to DA neurons by activated astrocytes. Astrocyte-mediated neuroinflammation and neuroprotective mechanisms in the pathogenesis of PD. Upon exposure to inflammatory stimuli, astrocytes become activated A1 phenotype. A1 phenotype astrocytes secrete pro-inflammatory factors such as IL-1β, TNF-α, and other inflammatory factors, which further induce neuroinflammation and neurotoxicity mechanisms in the human brain, damaging DAergic neurons. A1 phenotype astrocytes secrete pro-inflammatory factors such as IL-1β, TNF-α, and other inflammatory factors. On the other hand, the neuroprotective mechanism of activated A2 phenotype astrocytes against PD includes the release of anti-inflammatory cytokines into the brain as well as the upregulation of neuroprotective trophic factors, anti-inflammatory cytokines including TGF-β, which inhibit persistent neuroinflammation and thus protect DAergic neurons. Disease occurs when this balance in the organism is disrupted.

Activated astrocytes are an important component of the neuroinflammatory response in brain tissue and can be classified into A1 and A2 phenotypes, similar to microglia (77, 78, 64). A1-reactive astrocytes are widely seen in neurodegenerative diseases such as Alzheimer’s disease and PD and exhibit neurotoxic effects (77, 78). In contrast, the A2 phenotype has neuroprotective effects. A2-reactive astrocytes upregulate the expression of many neurotrophic factors to promote neuronal survival, growth, and differentiation (77, 79). A2-reactive astrocytes also upregulate the expression of anti-inflammatory cytokines, such as transforming growth factor beta (TGF-β), which are involved in synapse formation. However, the mechanism of conversion between the two phenotypes is currently unknown (77, 79). Activated astrocytes, the most abundant neuronal support cells in the human body, can also produce inflammatory cytokines such as IL-1β and TNF-α, while aggravating the damage to dopaminergic neurons and promoting the progression of LID (80). In mice, microglia can also promote the activation of astrocytes. Upon activation, the astrocyte nuclear factor kB (NF-kB) signaling pathway releases large amounts of TNF-α and IL-1β, which amplify the neuroinflammatory response and exacerbate dopaminergic neuronal damage (81). At the same time, high expression of glial fibrillary acidic protein (GFAP), cyclooxygenase-2 (COX-2), and iNOS increase TNF-α and IL-1β in cellular experiments and animal models (82), which can then activate astrocytes and microglia and trigger a neuroinflammatory response (83, 84). It is evident that astrocytes and inflammatory cytokines are closely related, and inflammatory cytokines have been shown to regulate the activation of astrocytes and microglia through positive feedback in animal models and in patients (85, 86). The morphology and spatial localization of mitochondria in astrocytes are affected by neuroinflammatory stimuli, which may result in excitotoxicity by interfering with the uptake of Ca^2+^-coupled Glu, affecting dopaminergic neuron survival and promoting the onset of LID. **Figure 2** depicts the mechanism of astrocyte damage to dopaminergic neurons.

Astrocytes can regulate intersynaptic D-type serine content, thereby regulating NMDAR activity and neurosynaptic plasticity (87, 88). Astrocyte proliferation via NMDAR signaling in neurons may play an important role in learning memory functions, neurodegenerative diseases, and psychiatric disorders (89, 90). Basic research has shown that astrocytes can sense the synaptic activity of neurons through membrane surface receptors and respond to information from neurons by altering intracellular Ca^2+^ concentrations and releasing gliotransmitters, Glu, ATP, and D-type serine, which regulate adjacent neurons and synaptic plasticity (91). There is also evidence that microglia affect astrocyte activity through ATP release (67). These findings from both *in vitro* and *in vivo* models suggest that astrocytes play a proactive role in the transmission of information between neurons (91, 92). D-type serine released from astrocytes had an active effect on NMDARs in cellular and animal experiments, especially on the GluN2A and GluN2B subunits (93). D-type serine released by astrocytes functions as a co-transmitter or co-agonist of NMDARs in mice (93). In addition, changes in Ca^2+^ concentration in astrocytes may also play an important role in the regulation of NMDAR activity in adjacent neurons (94). Glu enhances TNF-α secretion and GFAP expression in astrocytes and promotes the expression of ionized Ca^2+^-binding adapter molecule 1 (Iba-1) in microglia (95, 14). In experimental work on animal models, activated astrocytes release TNF-α, possibly through a self-reinforcing mechanism, which enhances the excitability and maintains astrocytes in an activated state, thus promoting LID (14, 95, 96). Therefore, astrocytes act on the development of LID through modulating NMDAR activation and expression, which in turn acts on synaptic plasticity in neurons.

## 6 Role of NMDARs and microglia-astrocyte interactions in LID

**Figure 3:**
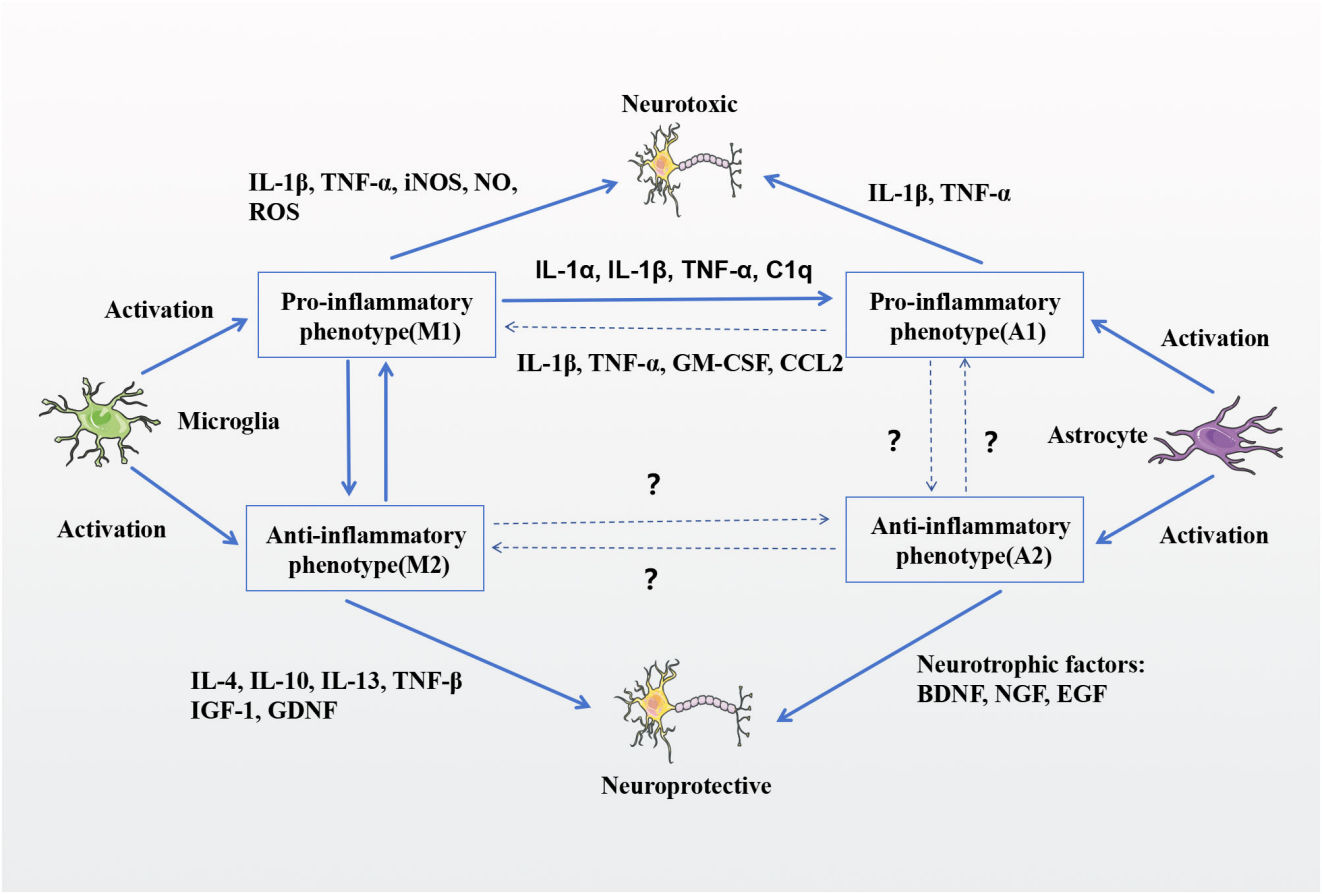
Interactions between microglia and astrocytes and mechanisms of damage to DA neurons. Both microglia and astrocytes have differentiated pro-inflammatory phenotypes that are neurotoxic and injurious to DAergic neurons, whereas the neuroprotective phenotypes are neuroprotective and protective of DAergic neurons. Under certain specific circumstances, the pro-inflammatory and anti-inflammatory phenotypes of microglia can be switched. Pro-inflammatory microglia secrete IL-1α, IL-1β, TNF-α, and complement component 1q (C1q), which can convert astrocytes to a pro-inflammatory phenotype. Pro-inflammatory astrocytes secrete IL-1β, TNF-α, Granulocyte-macrophage colony-stimulating factor (GM-CSF), and chemokine C-C motif ligand 2 (CCL2), which in turn activate pro-inflammatory microglia. The phenotypic transition of astrocytes remains to be clarified. Dashed lines with question marks indicate possible relationships, but evidence of a direct association is lacking.

Neuron**–**microglia**–**astrocyte interactions play a major role in synaptic plasticity in the neuronal response of postsynaptic neurons to L-DOPA (94, 97). Microglia and astrocytes are major players in the neuroinflammatory response, taking on a dual role between the immune and neuroinflammatory responses (98). The clinical development of LID relies on a cascade of altered pre- and postsynaptic messaging steps, leading to abnormalities in cortical neuronal messaging and abnormal changes in striatal projection neurons. In recent years, the inflammatory response induced by L-DOPA has been further explored. Both microglia and astrocytes express a variety of neurotransmitter receptors and regulate synaptic function at pre- and postsynaptic level by releasing a variety of soluble molecules. L-DOPA can over-activate glial cells, and the long-term presence of abnormally activated microglia and astrocytes leads to abnormal neuron–glia communication, which affects synaptic activity and neuroplasticity and exacerbates LID (14, 99). In response to external stimuli, such as aggregation of α-synuclein or LPS, microglia will rapidly transform into an activated state while releasing large amounts of inflammatory factors which, under inflammatory conditions at the site of injury, will further act on another type of glial cell—the astroglia (100). Stimulated astrocytes can activate and release inflammatory factors. These factors, as well as those released by microglia, act simultaneously on dopaminergic neurons and thereby lead to degeneration. Meanwhile, diseased neurons can release large amounts of toxic factors that continuously activate microglia, leaving the body in a state of marked inflammation. This positive feedback process ultimately exacerbates the development of LID both *in vivo* and *in vitro* (100, 101). In brain tissue of mouse models, it has been suggested that there are two main signals for microglia-mediated neurotoxicity: Glu and TNF-α (102). When Toll-like receptors (TLRs) stimulate microglia, they trigger pro-inflammatory programming, TNF-α production, and glutaminase expression, leading to increased Glu secretion (81, 103, 104). High levels of TNF-α and Glu can in turn induce neuronal death by stimulating NMDAR expression in neurons (105, 106). Thus, TNF-α and Glu act as two synergistic inflammatory mediators produced by microglia. TNF-α may also act on astrocytes to induce further production of TNF-α and other inflammatory molecules such as IL-6 and monocyte chemotactic protein 1 (MCP-1) (107). In addition, TNF-α inhibits the uptake of Glu by astrocytes, while astrocytes are the main mediators of Glu clearance in steady-state conditions (108). Abnormally increased Glu in the synaptic gap exacerbates LID by promoting NMDAR overexpression and altering synaptic plasticity (109, 110). **Figure 3** depicts the interaction of microglia and astrocytes and the mechanism of damage to dopaminergic neurons.

## 7 Role of NMDARs and cytokines in LID

Several studies have shown that fluctuating stimulation by L-DOPA will exacerbate inflammation in the striatum when dopamine is in a depleted state (3). The development of LID is accompanied by increased levels of IL-6, IL-12, IL-1β, TNF-α, iNOS, chemokines, NO, COX-2, and reactive oxygen species (ROS) in the dorsolateral striatum (97). Notably, IL-4, IL-10, and neuroprotective factors produced by glial cells are anti-inflammatory and play a very important role in maintaining glial cell and neuronal homeostasis (111). There is evidence that IL-4 and IL-10 can downregulate TNF-α and other pro-inflammatory cytokines and growth factors, which may reduce the occurrence of LID (14). Therefore, promoting the release of anti-inflammatory and neuroprotective factors *in vivo* will be a key research direction for the treatment of LID in the future. As mentioned above, there is increasing evidence that cytokines play a key role in the pathologic process of LID (12), and inhibition of neuroinflammation can downregulate NMDAR and thereby modulate the development of LID. The development of LID is inextricably linked to the activation of glial cells in the striatum and the release of inflammatory factors (TNF-α, IL-1β, and IL-6) (112). The development of neuroinflammation and the release of cytokines (IL-6, IL-12, IL-1β, TNF-α, iNOS, chemokines, NO, COX-2, and ROS) in brain tissues accelerate the development and progression of LID by activating the Glu2/PKC/MEK/ERK signaling pathway and the NF-κB signaling pathway (16).

**Figure 4:**
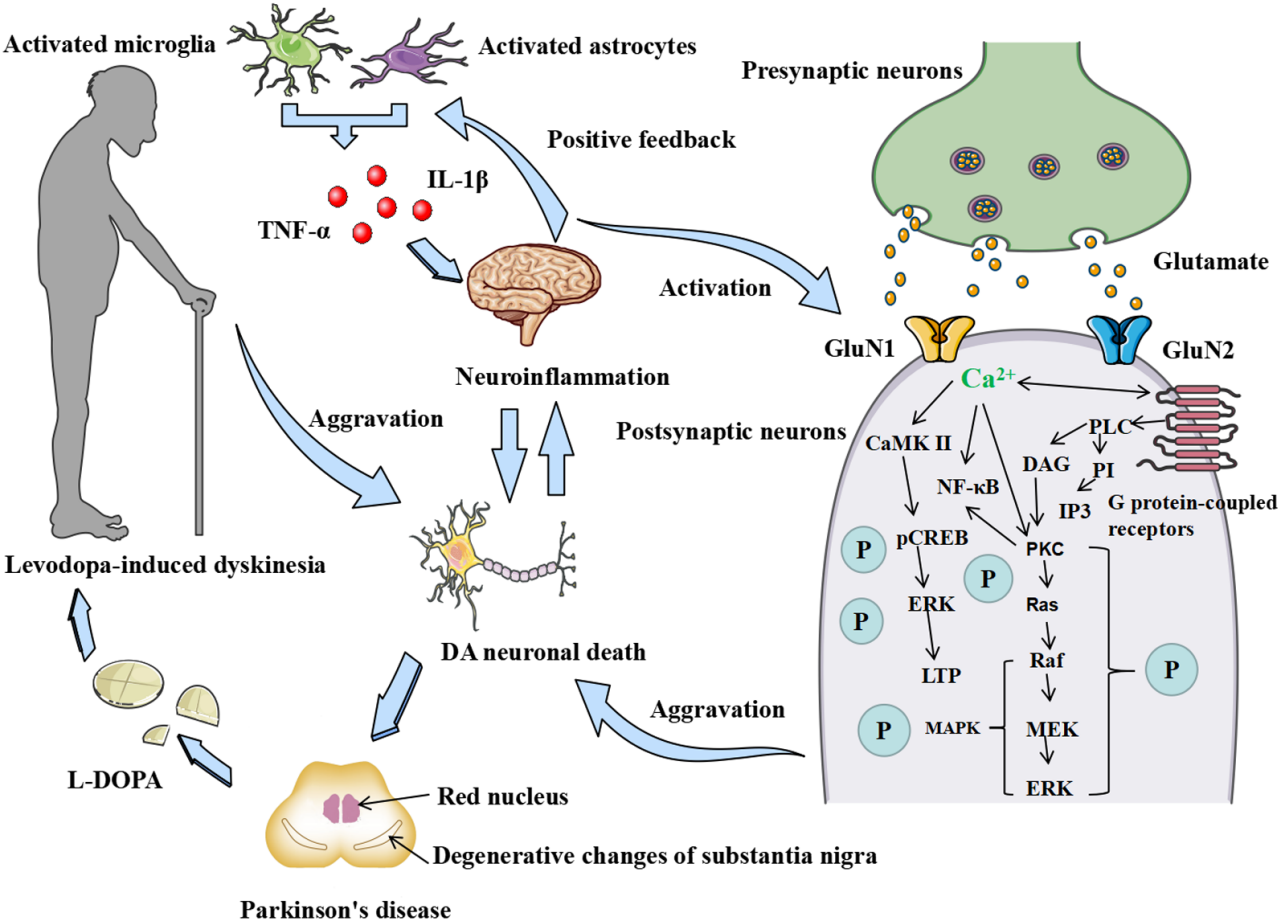
Correlations between neuroinflammation, NMDAR, and LID. Mutual activation of microglia and astrocytes and the release of inflammatory factors such as TNF-α and IL-1β. The release of inflammatory factors promotes the expression of the GluN1 and GluN2 subunits of the NMDAR in postsynaptic neurons, allowing the binding of excitatory neuronal Glu and NMDAR released from presynaptic neurons and the inward flow of Ga^2+^ to act on neuronal synaptic plasticity by regulating the LTP and MAPK/ERK phosphorylation pathways through calmodulin-dependent kinase II (CaMKII). The release of inflammatory factors, on the other hand, directly promotes the development of neuroinflammation in the brain, secondary to the death of DA neurons, which leads to the development of PD and the development of LID in patients with PD after years of L-DOPA administration by exacerbating the inflammatory response in the brain tissue and thus creating a vicious circle.

The accumulation of inflammatory factors in brain tissue exacerbates the activation of microglia and astrocytes, releasing more inflammatory factors and neurotransmitters, forming a vicious cycle. Microglia are essentially resident immune cells of the brain and nervous system (113). The release of Glu from microglia is a key component of neuronal damage (114, 115). Cytokines released by microglia, such as TNF-α, promote the release of Glu from astrocytes, which enhances excitotoxicity in neurons (116, 117). Once free Glu is increased in the brain, Glu receptors, such as NMDARs, are overstimulated, and large amounts of Ca^2+^ flow into the cells, thus leading to neuronal injury and death. The increased activity of Glu is thought to play an important role in the development of LID (118). LID may be attenuated by a decrease in glutamatergic function (118). **Figure 4** shows interactions of neuroinflammation and NMDARs and the possible sequence of events leading to LID.

## 8 Role of anti-inflammatory drugs and NMDAR antagonists in anti-LID

Thalidomide and 3,6’-dithiodide have anti-inflammatory effects and are thus able to reduce the neuroinflammatory response in the striatal region of the brain to a certain extent, including reducing abnormal involuntary movement (AIM) behavior in LID rats (11, 119). To date, no studies have confirmed that drugs with anti-inflammatory effects have a direct or indirect effect on NMDAR expression, and the only drug that shows a general clinical improvement in LID and is approved for clinical use is amantadine (120). Amantadine is a low-affinity, non-selective NMDAR antagonist that inhibits striatal NMDAR expression by reducing microglia proliferation, astrocyte GFAP expression, and cytokine release, thereby alleviating the onset of LID (121). Another NMDAR antagonist, ketamine, has recently been found to have a stronger binding affinity than amantadine (122, 123). Ketamine can regulate synaptic transmission and synaptic plasticity by inhibiting NMDAR activity, thereby restoring synaptic function in cortical and hippocampal regions caused by chronic stress. It also exhibits a neuroprotective effect during treatment, accompanied by increased microglia phagocytosis and increased anti-inflammatory factor IL-6 in the striatum, which suggests low-dose ketamine as a potential treatment for LID (124, 125, 126, 123). In addition, the treatment of simvastatin improves cognitive function, anxiety, and depression in MPTP-treated LID mice, restores nerve growth factor IB (Nur77) downstream by reversing the increase in GluN2B expression, and reduces COX-2 and TNF-α (127, 128). A new experimental drug, agmatine, can improve the behavior and AIM scores of LID rats by inhibiting NMDAR expression in the substantia nigra, thereby suppressing the inflammatory and oxidative stress cascades, activating erythroid 2-related factor 2 (Nrf2), and inhibiting the NF-κB signaling pathway to improve the antioxidant, anti-inflammatory, and anti-apoptotic properties of LID (129, 130).

Except for amantadine, there is no medical evidence to suggest that drugs like ketamine, simvastatin, or guanfacine can induce further improvement in neuroinflammation and LID after NMDAR antagonization. Amantadine is therefore the only drug currently used for the clinical treatment of LID. A clinical cohort might help explore the selective use of the aforementioned anti-LID drugs and provide more clinical evidence of their efficacy against LID. Further drugs that can antagonize NMDAR need to be identified, and the mechanisms underlying the relationships between NMDAR, neuroinflammation, and LID need to be examined to optimize the treatment of patients with LID. **Table 1** showcases the main references and findings that demonstrate the connection between LID, NMDARs, and neuroinflammation.

**Table 1:** Study characteristics. It showcases the main references and findings that demonstrate the connection between LID-NMDAR-neuroinflammation.

Author	Year	Country	Type of Study	Designof Study	Results
Angelopoulou et al.	2021	Greece	_	Review	Fyn kinase may regulate LID, enhanced neuroinflammation and glutamate excitotoxicity by mediating NMDAR axes.
Azar et al.	2022	Egypt	Rat	RCT	Agmatine-mediated inhibition of NMDAR expression and amelioration of dyskinesia with a focus on its anti-inflammatory potentiality.
Bartlett et al.	2020	USA	Rat	RCT	Ketamine is an NMDAR antagonist.The long-term effects of ketamine depend on BDNF signaling in the striatum. And it attenuates the development of LID in rodents.
Bortolanza et al.	2015	Brazil	Rat	RCT	Glu are bound to influence microglial activation states. And LID leads to upregulation of iNOS, GFAP and OX-42-ir.
Carta et al.	2017	Italy	_	Review	LID and neuroinflammation: microglia and astrocytes play a key role.
Cerovic et al.	2015	Italy	Mice	RCT	The Ras-ERK signaling has a central role in striatal LTP, depotentiation, and LTP restored after L-DOPA treatment.
Chen et al.	2022	China	Rat	RCT	Neuroinflammation caused sustained downregulation of synaptic NR2A and NR2B subunits. And anti-inflammatory treatment reversed the downregulation and hypofunction of synaptic NR2A and NR2B.
Gurrera	2019	USA	Human	RCT	Patients with anti-NMDA receptor encephalitis can develop motor dysfunction.
Innes et al.	2019	UK	_	Review	Changes to synaptic plasticity may be mediated by microglial modifications. Microglial production of cytokines may regulate LTP and LTD, thereby underlying the development of disease.
Koh et al.	2022	South Korea	Mice	RCT	Astrocytes regulate NMDAR tone via BEST1-mediated corelease of D-serine and Glu.
Morissette et al.	2022	Canada	Monkeys	RCT	Increased inflammatory markers in the basal ganglia associated with LID and revealed that MPEP inhibition of glutamate activity reduced LID and levels of inflammatory markers.
Pereira et al.	2021	Brazil	Mice	RCT	The release of TNF-α by glutamate-activated astrocytes may contribute to LID by exacerbating corticostriatal glutamatergic inputs excitability and maintaining astrocytes in an activated state through a self-reinforcing mechanism.
Rahman et al.	2022	Australia	Human	RCT	Neuroinflammation may alter NMDAR stoichiometry, and future studies could aim to determine if anti-inflammatory treatment can alleviate this aspect of NMDAR-related pathology.
Rentsch et al.	2020	Australia	Mice	RCT	Amantadine, an NMDAR antagonist, may explore novel features of microglia and astrocyte physiology and pathophysiology and their direct and/or indirect impact on neuronal synaptic signalling in LID.
Thiele et al.	2014	Canada	Mice	RCT	In the LID state, the direct pathway exhibits only LTP, becoming generally overactive, and the indirect pathway exhibits only LTD.
Trudler et al.	2021	California	Mice	RCT	Glutamate release from astrocytes and excessive extrasynaptic NMDAR activity in neurons, thus contributing to Synapse and neuron Loss.
Varley et al.	2019	UK	Human	RCT	The Movement disorder associated with NMDAR antibody-encephalitis is complex and characteristic.
Wang et al.	2018	China	Rat	RCT	CaMKIIa-GluN2B interaction had an important role in the development of LID. CaMKII is also associated with inflammatory pathways.
Yan et al.	2021	China	Rat	RCT	Systemic inflammation increases the susceptibility to LID in 6-OHDA lesioned rats by targeting the NR2B-Medicated PKC/MEK/ERK Pathway.
Yuan et al.	2023	China	Rat	RCT	Interventions targeting astrocytes and glutamate transporters may delay LID.

( Randomized Controlled Trial: RCT. ).

## 9 Summary and outlook

The regular release of dopamine in brain tissue starts fluctuating after long-term administration of L-DOPA to patients with PD. These fluctuations lead to changes in receptors on a variety of neurons in the striatum involved in intracellular information transmission, synaptic plasticity, and other processes, which further disrupt the already unbalanced basal ganglia system. This results in a progressive impairment of motor control and, ultimately, the development of LID. There is a lack of research into the mechanisms underlying LID and associated potential treatment approaches. Excessive Glu, exerting neurotoxic effects, has been found to be closely related to LID, and NMDAR plays a major role in the pathogenesis of LID due to its high Ca^2+^ permeability, abnormal subunit expression, and phosphorylation levels. In recent years, neuroinflammation has emerged as a hot topic in the study of the pathogenesis of LID. In response to external stimuli, glial cells can be activated into pro- and anti-inflammatory phenotypes, and pro-inflammatory microglia and astrocytes play an important role in neuroinflammation, which in turn increases the expression of striatal NMDAR GluN1 or GluN2 subunits and activates phosphorylation pathways, thereby exacerbating LID. Anti-inflammatory microglia and astrocytes, on the other hand, release anti-inflammatory cytokines and neuroprotective factors that slow down the progression of LID, and the conversion of the two may be a new direction of research for the treatment of LID in the future. The activation of microglia and astrocytes, the interconversion of the two, and the release of neuroinflammatory factors such as TNF-α and IL-1β can directly promote dopaminergic neuronal death as well as stimulate the release of Glu. Both microglia and astrocytes contribute to NMDAR overexpression in postsynaptic neurons and exacerbate LID by mediating LTP and the MEK/ERK/MAPK phosphorylation pathways and altering synaptic neuronal plasticity.

Currently, the most common anti-LID drug is amantadine. While ketamine, simvastatin, guanfacine, and other drugs still have a certain effect on LID, there is a lack of relevant clinical evidence. The development of new drugs and clinical translational applications targeting inflammatory factors, NMDARs, and LID will benefit patients who develop AIM after clinical treatment with L-DOPA drugs, improve their quality of life, and reduce the burden on society.

## Author contributions

FZ and ML contributed to the conception and design of this study and collected and reviewed the relevant literature. FZ and ML designed the article structure. FZ wrote the first draft of this manuscript. All authors contributed to the manuscript revision and read and approved the submitted version.
